# Do Dynamic Plantar Pressures Differ Based on Sonographic Evidence of Metatarsophalangeal Joint Synovitis in People With Rheumatoid Arthritis?

**DOI:** 10.1002/acr2.11635

**Published:** 2023-12-20

**Authors:** Libby Anderson, Belinda Ihaka, Catherine Bowen, Charlotte Dando, Sarah Stewart

**Affiliations:** ^1^ Auckland University of Technology Auckland New Zealand; ^2^ Active Living and Rehabilitation, Aotearoa, and Health and Rehabilitation Research Institute, School of Clinical Sciences, Auckland University of Technology Auckland New Zealand; ^3^ University of Southampton Southampton United Kingdom

## Abstract

**Objective:**

The metatarsophalangeal joints (MTPJs) are the most common location for synovitis in people with rheumatoid arthritis (RA), yet their association with plantar foot pressures has received very little attention. This study aimed to determine whether plantar pressures differed based on sonographic evidence of MTPJ synovitis in people with RA.

**Method:**

Ultrasound was used to assess synovitis (grey scale synovial hypertrophy and power Doppler signal) in MTPJs 1 to 5 using the combined EULAR/Outcome Measures in Rheumatology scoring system. Peak pressure (PP) and pressure time integrals (PTIs) were assessed during barefoot walking for seven plantar foot regions (heel, midfoot, first metatarsal, second metatarsal, third to fifth metatarsals, hallux, lesser toes). Mixed‐effects linear regression was used to determine the difference in PP and PTI between MTPJs with none/minimal synovitis and MTPJs with moderate/severe synovitis.

**Results:**

Thirty‐five participants with RA were included. Mean age was 66.3 years and mean disease duration was 22.2 years. Participants with sonographic evidence of moderate/severe synovitis at the first MTPJ had reduced PTI at the hallux compared with those with none/minimal synovitis at this joint (*P* = 0.039). Participants with moderate/severe synovitis at the second MTPJ and fourth MTPJ had reduced PP and reduced PTI at lesser toes compared with those with none/minimal synovitis in these joints (all *P ≤* 0.048). No significant differences were observed for synovitis in other joints.

**Conclusion:**

These findings may be suggestive of an inverse relationship between plantar pressure and soft tissue pathology, which is consistent with an offloading strategy and reduced use of the toes during propulsion.

## INTRODUCTION

Inflammation targeting synovial joints (synovitis) and surrounding soft tissue structures is central to the pathophysiology of rheumatoid arthritis (RA) and a major source of patient‐reported pain.^1^ The most common site of synovitis is the metatarsophalangeal joints (MTPJs) in the forefoot.^2^, ^3^ Chronic inflammation in this region contributes to digital deformities, including hallux valgus, clawed toes, subluxation, and plantar prominence of the metatarsal heads.^4^ As a result, the plantar MTPJs may be prone to mechanical overload, which further exacerbates pain and discomfort during weight‐bearing activity.^5^, ^6^

Research has shown that people with RA exhibit altered plantar pressure patterns during barefoot walking when compared with people without RA.^7^ Both peak pressure (PP) and pressure time integrals (PTIs) have been identified as important measures in people with RA.^8^ This is represented by the highest pressure experienced in a specific region in the foot (PP) and the length of time that pressure is present (PTI).^8^ However, region‐specific pressure differences between studies vary considerably.^4^, ^6^, ^9^, ^10^ Increased plantar pressures in the forefoot in people with RA have been associated with pain, digital deformities, and radiographic evidence of bone erosion.^5^, ^6^ Contrastingly, lower forefoot pressures in people with severe RA have been associated with greater functional incapacity and discomfort.^10^ The results from these studies suggest that the biomechanical impact of RA on the foot is complex and likely determined by a combination of compensatory offloading mechanisms and underlying inflammation and structural damage.

Synovitis, an important marker of disease activity, has been observed in people with RA in the absence of clinical evidence of joint inflammation.^11^, ^12^ Subclinical synovitis (ie, not detected by methods of clinical assessment) is frequently evaluated using ultrasound imaging, which visualizes enlargement of the synovium through grey scale synovial hypertrophy, as well as increased microvascular blood flow using power Doppler capability.^13^ The presence of the power Doppler signal in the rearfoot (subtalar joint) has been associated with reduced medial and lateral heel plantar pressures in people with RA, which the authors attribute to a pain‐avoidance gait strategy.^14^ The presence of a combined score of synovial hypertrophy and bone erosion in the forefoot has also been associated with reduced PPs beneath the lateral forefoot in people with RA^15^; however, it is unclear whether MTPJ synovitis alone, which is present in early disease stages before osseous damage develops, influences plantar pressure.

Despite the MTPJs being the most common location for synovitis in the foot in people with RA,^2^, ^3^ the relationship between ultrasound evidence of MTPJ synovitis and plantar pressure has not been investigated. We hypothesize that pressure at various regions of the plantar foot during weight‐bearing activity may differ depending on whether sonographic evidence of MTPJ synovitis is present. This information will provide us with an increased understanding of the foot‐ground interface pressures in people with RA. The aim of this study was to investigate whether dynamic plantar pressures (PP and PTIs) differed based on sonographic evidence of MTPJ synovitis in people with RA.

## PATIENTS AND METHODS

### Study design

This cross‐sectional observational study was conducted in accordance with the EULAR recommendations for the reporting of ultrasound studies in rheumatic and musculoskeletal diseases.^16^

### Participants

Participants were recruited through public advertising via Arthritis New Zealand and the Auckland University of Technology (AUT) Podiatric Rheumatology Clinic (Auckland, New Zealand). Participants were included if they had a physician‐diagnosis of RA and met the American College of Rheumatology Classification Criteria for RA,^17^ were able to walk barefoot, and aged over 20 years. The exclusion criteria included people with other inflammatory rheumatic disorders (eg, crystal arthropathies, spondylarthritis, systemic lupus erythematosus, etc.). Ethical approval was obtained from the AUT Ethics Committee (AUTEC 22/261). All participants were required to provide written informed consent prior to data collection.

### Data collection

All participants attended a single study visit at the AUT North Campus (Northcote, Auckland) or the AUT South Campus (Manukau, Auckland). Demographic and clinical characteristics were recorded for all participants on a standardized clinical report form, including, age, sex, self‐reported ethnicity (NZ European, Māori, Pacific Peoples, Middle Eastern, Hispanic, African American, Asian, Other), RA disease duration, medications, and comorbidities. Participants were also asked to complete a 100 mm foot pain visual analog scale (VAS), indicate any areas of pain on a Chatterton Foot Diagram,^18^ and complete the Rheumatoid Arthritis Foot Disease Activity Index (RADAI‐F5). The RADAI‐F5 is a reliable and valid measures of foot disease activity in people with RA.^19^ It contains five questions related to foot disease activity in which participants rate from 0 to 10. These scores are then summated and divided by five to provide an average overall score in which mild disease activity is greater than 1 to 3.6, moderate disease activity is greater than 3.6 to 5.7, and high disease activity is greater than 5.7 to 10. Height and weight were measured to calculate body mass index (BMI), and specific foot joints were examined for the presence of palpable tenderness and swelling (MTPJs 1–5, digital interphalangeal joints 1–5, midtarsal joint, subtalar joint, talocrural joint).

#### Dynamic plantar pressure

A dynamic assessment of plantar pressure of both feet was performed using a 5‐mm‐thick pressure mat (432 mm × 368 mm; TekScan). The mat incorporates 2,288 resistive sensors that sample at a rate of 40 Hz. Prior to data collection, the mat was calibrated to the participant's weight. The two‐step gait initiation protocol was used to ensure that each participant's foot contacted the sensor area of the mat with the second step.^20^ Participants were instructed to walk barefoot across the mat at their own comfortable walking pace. Two to three practice walking trials were completed to familiarize participants with the procedure and to facilitate a normal walking pattern across the mat. Following this, three trials per left and right foot were recorded. The TekScan software was used to mask each foot into seven regions of interest (ROI) representing the hallux (ROI1), lesser toes (ROI2), metatarsal one (ROI3), metatarsal two (ROI4), metatarsals three to five (ROI5), the midfoot (ROI6), and the heel (ROI7). Manual adjustments and corrections were made to the masking as appropriate to ensure the most optimal position to represent the anatomical structure of the plantar surface of the foot. This masking method has demonstrated excellent reliability for the calculation of pressure measurements during barefoot level walking.^21^ Following generation of the masking template for each foot, the peak plantar pressure (kPa), and PTIs (kPa*sec) were calculated for each ROI.

#### Ultrasound assessment

Prior to data collection, the primary researcher (LA) undertook approximately 40 hours of training under a podiatrist with more than 10 years of experience in ultrasound assessment of the foot (BI). As part of this training, a reliability exercise was undertaken to ensure the researcher was competent in performing the scans. A Logique‐e (GE Healthcare) ultrasound machine with a wide‐band array transducer (4.2–13.0 MHz) was used. Assessment of bilateral MTPJs was performed in accordance with the EULAR guidelines for musculoskeletal ultrasound in rheumatology.^22^ Participants were positioned seated with their legs extended. A water‐based gel was applied to the plantar forefoot and the MTPJs were scanned in the longitudinal plane. The joints were maximally plantarflexed (if adequate joint motion was available) to optimize visualization of the joint space. Power Doppler parameters in B‐mode using the factory settings were adjusted to maximize sensitivity by decreasing the pulse repetition frequency and wall filters and adjusting the Doppler gain to just below the level at which color noise disappears in the cortical bone (no flow should be visualized at the bony surface). Scans took approximately 25 minutes per participant. Static images were saved for each of the MTPJs on right and left feet for later grading.

#### Ultrasound grading

Joints were scored for grey scale synovial hypertrophy and power Doppler using a semiquantitative scoring system ranging from 0 to 3 (0 = none, 1 = minimal, 2 = moderate, and 3 = severe).^23^ These scores were then combined to produce an overall score for synovitis in accordance with the EULAR/Outcome Measures in Rheumatology combined scoring system, which recognizes the relative importance of both components in defining synovitis.^23^ Grade 1 (minimal synovitis) is defined as synovial hypertrophy = 1 and power Doppler less than or equal to 1. Grade 2 (moderate synovitis) is defined as synovial hypertrophy = 2 and power Doppler less than or equal to 2, or synovial hypertrophy = 1 and power Doppler = 2. Grade 3 (severe synovitis) is defined as synovial hypertrophy = 3 and power Doppler less than or equal to 3, or synovial hypertrophy = 1 or 2 and power Doppler = 3. All images were graded independently by two readers (BI, SS), blinded to the participants and all other data. The two readers reached agreement on 263 out of 350 images (75.1%). The two readers then met to discuss the remaining discrepancies in order to agree on a final score for each image. Images in which either synovial hypertrophy or power Doppler could not be graded (ie, because of poor visibility of the joint space) were excluded. For the purpose of data analysis, the semiquantitative scores for all included images were dichotomized by grouping grade 0 (none) and grade 1 (minimal) into “none/minimal synovitis” and by grouping grade 2 (moderate) and grade 3 (severe) into “moderate/severe synovitis.” This method was chosen because of the high level of subjectivity in differentiating between grades 0 and 1, the frequent presence of grade 1 in healthy populations,^24^ and grade 2 or more being considered more reflective of definite pathological synovitis at the MTPJs in people with RA.^25^, ^26^

#### Inter‐ and intra‐rater reliability for ultrasound grading

To determine inter‐reader reliability, a random 10% of images (n = 35) were scored by consensus between two additional readers (CB, CD) who were blinded to the scores of the first two readers. Intra‐rater reliability was also determined using the same random set of images that were rescored approximately 4 months later by the original readers (BI, SS) who were blinded to their original scores. Intra‐ and inter‐reader reliability were calculated for the original semiquantitative scoring (grades 0 to 3) for power Doppler and synovial hypertrophy.

### Data analysis

Descriptive statistics were reported as mean (SD) for continuous data and n (%) for categorical data. Linear regression models were used to determine whether plantar pressure values (continuous variables of peak plantar pressure and PTIs at the seven ROIs) significantly differed between none/minimal synovitis and moderate/severe synovitis for each MTPJ (dichotomous variable). The distribution of residuals for each linear model was assessed prior to inferential analyses to ensure sufficient normality was present. To account for the dependence between right and left feet, and the dependence between the seven plantar pressure ROIs (which form a natural vector of related variables), a mixed‐effects approach was adopted.^27^ This involved the inclusion of a participant‐specific random effect and participant‐nested random effect for foot side to account for repeated right and left foot measures. To address the association between the seven masked plantar pressure ROIs, a heterogenous compound symmetry covariance structure on the model residuals was used. This allowed separate variances for each plantar region, as well as different covariances, between each pair of regions. All models were also adjusted for BMI because of its influence on plantar pressure. This model allows for reweighting because of missing values and accommodates missingness without further adjustment.

To determine inter‐rater reliability of both the original semiquantitative grading of synovial hypertrophy and power Doppler (grades 0 to 3), two‐way random, single measures, absolute agreement intraclass correlation coefficients ICC(_2,1_) were used. Similarly, to determine intra‐rater reliability, two‐way mixed, single measures, absolute agreement ICC_(3,1)_ were used. ICCs and their 95% confidence intervals (95% CIs) were reported and interpreted using the following benchmarks: less than or equal to 0.30 no agreement; 0.31 to 0.50 weak agreement; 0.51 to 0.70 moderate agreement; 0.71 to 0.90 strong agreement; greater than or equal to 0.91 very strong agreement.^28^

All hypothesis tests were carried out at a 5% level of significance against two‐sided alternatives. No adjustments were made for multiplicity because of the exploratory nature of this study,^29^ but all test statistics (least‐squares means), their null distributions, and their observed significance levels were reported. No adjustments were made for ethnicity. Data were analyzed using Statistical Analysis System (SAS) 9.4 and Statistical Package for the Social Sciences (SPSS) v25.

### Sample size calculation

Using the Power Analysis Sample Size 15 software and a repeated measures analysis, an estimated 35 participants would allow the detection of a difference in average peak plantar pressure between two groups (expected means: synovitis absent = 131 N/cm^2^, synovitis present = 40 N/cm^2^), with 80% power and a type I error rate of 0.05. This is based on an F Test with a two‐level within‐subject factor (left foot, right foot), a two‐level between‐subject (ultrasound synovitis present, ultrasound synovitis absent), a between‐subject SD of 100 N/cm^2^, and an autocorrelation among the two repeated measurements of 0.6. Calculations were based on expected means and SD for peak plantar pressure reported in previous work.^14^

## RESULTS

### Participant demographic and clinical characteristics

A total of 35 participants with RA were included in the study. The majority of participants were European (91%), were women (86%), and had a mean age of 66 years (Table 1). Many participants were taking multiple medications, with two‐thirds taking disease modifying antirheumatic drugs. The participants also reported other comorbid conditions, with hypertension and cardiovascular disease being the most common.

**Table 1:** Participant demographic and clinical characteristics*

N	35
Age, mean (SD), y	66.3 (13.4)
Ethnicity, n (%)	
New Zealand European	32 (91)
Asian	2 (6)
Hispanic	1 (3)
Sex, n (%)	
Female	30 (86)
Male	5 (14)
BMI, mean (SD)	27.1 (6.8)
RA disease duration, mean (SD), y	22.2 (15.2)
Medications, n (%)	
DMARDs	23 (66)
NSAIDs	11 (31)
Steroids	5 (14)
Biologic agents	4 (11)
Opiates	1 (3)
Antiplatelets	8 (23)
Anticoagulants	5 (14)
Antihypertensives	11 (31)
Hypoglycemics	1 (3)
Psychotropics	8 (23)
Comorbidities, n (%)	
Hypertension	8 (23)
Cardiovascular disease	6 (17)
Depression	5 (14)
Asthma	2 (6)

* BMI, body mass index; DMARD, disease modifying antirheumatic drug; NSAID, nonsteroidal antiinflammatory; RA, rheumatoid arthritis.

### Participant foot characteristics

**Figure 1:**
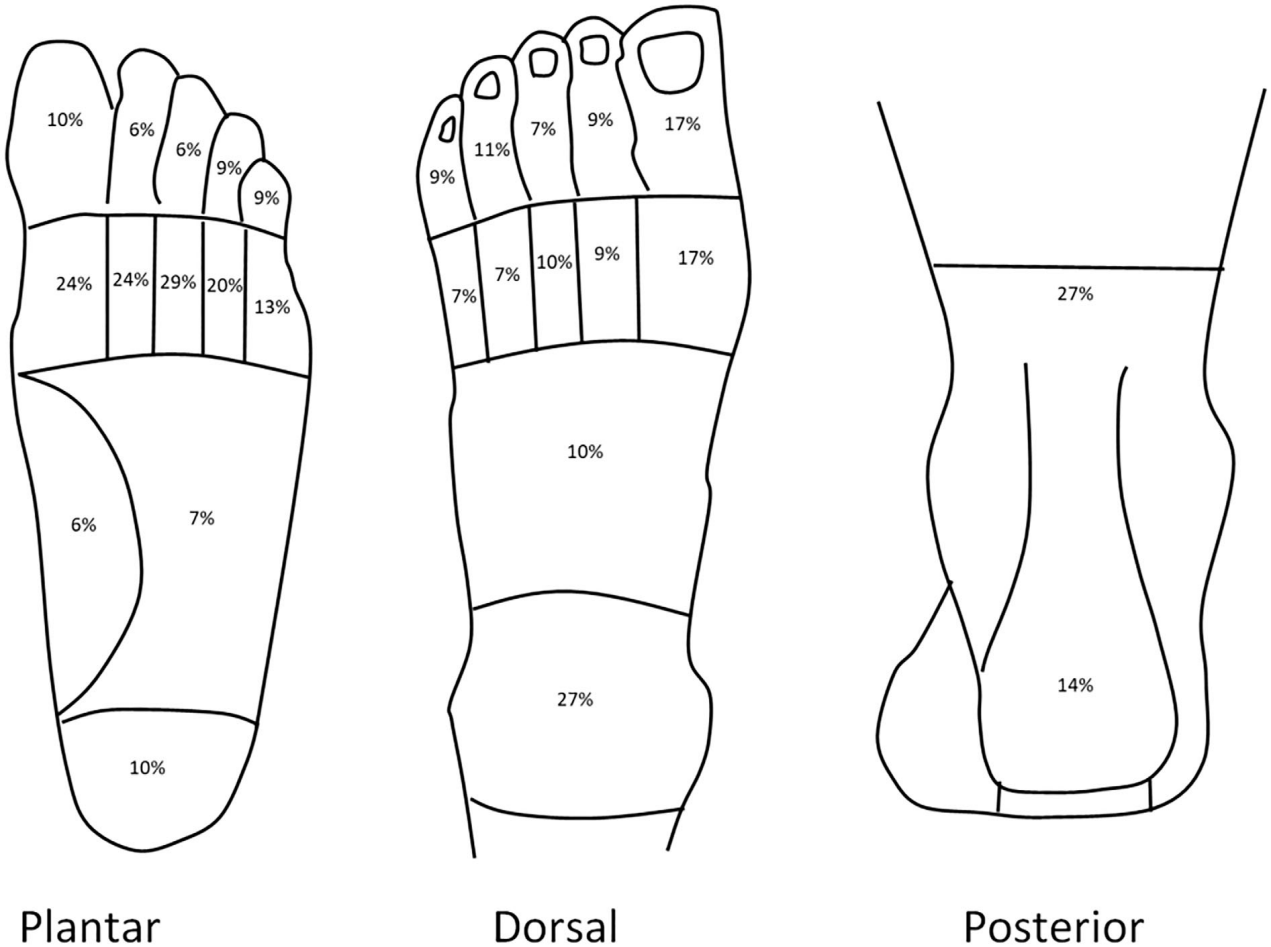
Patient‐reported pain locations according to Chatterton Foot Pain Diagram. Percentages calculated from number of feet (n = 70).

Foot‐specific characteristics are presented in Table 2. The mean patient‐reported foot pain VAS was 36.7 mm (SD 23.9) indicating a moderate level of pain, whereas the mean RADAI‐F5 score was 3.17 (SD 2.39), indicating mild disease severity. Researcher‐identified palpable tenderness was observed across all locations of the foot and ankle, with the midtarsal joint and central MTPJs being the most common locations. Researcher‐identified swelling was most often noted in the ankle and first MTPJ. Analysis of the Chatterton Foot Diagram showed that participants reported pain across all locations of the foot, with pain most often felt in the ankle and the plantar metatarsal region (Figure 1).

**Table 2:** Participant foot characteristics*

Characteristics		
100 mm VAS for foot pain, mean (SD)	36.7 (23.9)	
RADAI‐F5, mean (SD)	3.17 (2.39)	

Palpable tenderness and swelling, n (%)^a^	Tender joints	Swollen joints
Ankle	9 (13)	6 (9)
Subtalar joint	9 (13)	0 (0)
Midtarsal joint	23 (33)	0 (0)
First MTPJ	10 (14)	5 (7)
Second MTPJ	15 (21)	3 (4)
Third MTPJ	18 (26)	3 (4)
Fourth MTPJ	22 (31)	2 (3)
Fifth MTPJ	11 (16)	2 (3)
Hallux	5 (7)	3 (4)
Second toe	15 (21)	1 (1)
Third toe	12 (17)	0 (0)
Fourth toe	14 (20)	0 (0)
Fifth toe	11 (16)	0 (0)

* ^a^Percentages calculated from number of feet (n = 70).

MTPJ, metatarsophalangeal; RADAI – F5, rheumatoid arthritis disease activity index – foot five; VAS, visual analog scale.

### Inter‐ and intra‐reader reliability

Inter‐rater reliability for grey scale synovial hypertrophy was moderate (ICC_2,1_ = 0.52 [95% CI 0.21–0.73]) and power Doppler was strong (ICC_2,1_ = 0.73 [95% CI 0.51–0.85]). Intra‐rater reliability for grey scale synovial hypertrophy was strong (ICC_3,1_ = 0.80 [95% CI 0.58–0.90]) and power Doppler was very strong (ICC_3,1_ = 0.95 [95% CI 0.91–0.98]).

### Association between synovitis and plantar pressure

In total, 350 individual ultrasound images were obtained across the 35 included participants. Eight (2.3%) images from participants with joint deformity were excluded because of poor visualization of the joint space. The proportion of images with none, minimal, moderate, and severe power Doppler and synovial hypertrophy grades are shown in Table 3. Based on the dichotomization of the combined EULAR/Outcome Measures in Rheumatology scores for synovitis, 40.9% (n = 140) of joints displayed none/minimal synovitis, and the remaining 59.1% (n = 202) of joints displayed moderate/severe synovitis.

**Table 3:** Proportion of feet with ultrasound evidence of power Doppler, synovial hypertrophy and combined EULAR/OMERACT synovitis*

	First MTPJ	Second MTPJ	Third MTPJ	Fourth MTPJ	Fifth MTPJ
Power Doppler grade, n (%)					
n^a^	70	70	70	70	70
0 (none)	47 (67)	48 (69)	50 (71)	42 (60)	53 (76)
1 (minimal)	19 (27)	15 (21)	13 (19)	19 (27)	13 (19)
2 (moderate)	4 (6)	7 (10)	7 (10)	9 (13)	4 (6)
3 (severe)	0 (0)	0 (0)	0 (0)	0 (0)	0 (0)
Synovial hypertrophy grade, n (%)					
n^a^	67	69	68	70	68
0 (none)	18 (27)	10 (14)	6 (9)	5 (7)	2 (3)
1 (minimal)	25 (37)	20 (29)	17 (25)	25 (36)	18 (26)
2 (moderate)	17 (25)	29 (42)	36 (53)	31 (44)	34 (50)
3 (severe)	7 (10)	10 (14)	9 (13)	9 (13)	14 (21)
Combined EULAR/OMERACT grade for synovitis, n (%)					
n^a^	67	69	68	70	68
0 (none)	12 (18)	5 (7)	2 (3)	4 (6)	2 (3)
1 (minimal)	30 (45)	24 (35)	20 (29)	24 (34)	17 (25)
2 (moderate)	18 (27)	30 (43)	37 (54)	33 (47)	35 (51)
3 (severe)	7 (10)	10 (14)	9 (13)	9 (13)	14 (21)
Dichotomized scoring of synovitis^b^					
n^a^	67	69	68	70	68
None/minimal	42 (63)	29 (42)	22 (32)	28 (40)	19 (28)
Moderate/severe	25 (37)	40 (58)	46 (68)	42 (60)	49 (72)

* ^a^Refers to number of images included for analysis (some images excluded due to poor visibility of joint space).

^b^ Synovitis scores used in inferential analyses.

MTPJ, metatarsophalangeal joint; OMERACT, Outcome Measures in Rheumatology.

#### Peak plantar pressure

The regression results comparing ultrasound synovitis with peak plantar pressure are shown in Table 4. Participants with moderate/severe synovitis at the second MTPJ and fourth MTPJ displayed significantly reduced peak plantar pressure beneath the lesser toes compared with those with none/minimal synovitis in these joints (*P* = 0.048 and *P* = 0.018, respectively). No other significant differences in peak plantar pressure were observed for synovitis in other joints.

**Table 4:** Difference in peak plantar pressure (kPa) between none/minimal synovitis and moderate/severe synovitis according to the combined EULAR/OMERACT scores*

	Grade	First MTPJ mean	Diff (95% CI)	*P*	Second MTPJ mean	Diff (95% CI)	*P*	Third MTPJ mean	Diff (95% CI)	*P*	Fourth MTPJ mean	Diff (95% CI)	*P*	Fifth MTPJ mean	Diff (95% CI)	*P*
Heel	None/minimal	281.21	35.44 (9.95 to 80.82)	0.12	298.57	−5.29 (−50.29, 39.71)	0.82	300.44	–10.58 (56.72 to 35.55)	0.65	300.57	–9.69 (52.90 to 33.52)	0.66	279.97	20.84 (29.24 to 70.92)	0.41
	Moderate/severe	316.65			293.29			289.86			290.88			300.81		
Midfoot	None/minimal	188.41	–7.44 (55.30 to 38.43)	0.75	183.09	0.16 (‐43.98, 44.31)	0.99	187.36	–1.76 (47.78 to 44.26)	0.94	183.03	1.68 (40.47 to 43.83)	0.94	189.27	–8.91 (57.05 to 39.22)	0.71
	Moderate/severe	180.97			183.25			185.60			184.71			180.35		
First metatarsal	None/minimal	254.76	–15.89 (69.13 to 37.34)	0.55	263.91	−22.03 (‐74.92, 30.85)	0.41	242.27	9.64 (43.64 to 62.91)	0.72	242.89	10.84 (41.30 to 62.99)	0.68	263.15	–17.60 (76.61 to 41.42)	0.55
	Moderate/severe	238.87			241.88			251.91			253.73			245.56		
Second metatarsal	None/minimal	295.43	–38.27 (92.52 to 15.99)	0.16	276.29	7.79 (−45.00, 60.58)	0.77	263.39	26.34 (29.59 to 82.26)	0.35	279.29	–1.41 (53.31 to 50.49)	0.96	312.10	–42.51 (99.79 to 14.77)	0.14
	Moderate/severe	257.17			284.08			289.72			277.88			269.60		
Third to fifth metatarsals	None/minimal	292.93	–9.36 (56.62 to 37.90)	0.69	287.88	3.00 (−42.82, 48.82)	0.90	282.31	5.18 (41.26 to 51.63)	0.82	282.84	9.87 (34.27 to 54.01)	0.65	291.81	–3.69 (54.70 to 47.33)	0.88
	Moderate/severe	283.57			290.88			287.49			292.71			288.12		
Hallux	None/minimal	216.61	–47.60 (100.89 to 5.68)	0.08	221.45	–45.50 (97.18 to 6.18)	0.08	214.36	–29.81 (83.50 to 23.89)	0.27	197.98	–7.46 (59.54 to 44.61)	0.78	199.04	–15.38 (71.10 to 40.34)	0.58
	Moderate/severe	169.01			175.95			184.56			190.52			183.66		
Lesser toes	None/minimal	123.61	–29.26 (66.26 to 7.74)	0.12	132.00	–34.74 (69.17 to –0.32)	**0.048**	119.25	–12.81 (49.38 to 23.77)	0.48	135.39	–39.85 (72.44 to –7.26)	**0.018**	118.61	–11.59 (51.08 to 27.90)	0.56
	Moderate/severe	94.35			97.26			106.45			95.54			107.02		

* *Source:* Results are presented adjusted for BMI. Bolded ***P*** values indicate significant differences in pressure time integrals between combined EUALR/OMERACT grades as *P* < 0.05. None/minimal synovitis = grade 0 or 1. Moderate/severe synovitis = grade 2 or 3. Mean = least‐squares mean. Results are unadjusted for ethnicity.

Diff, difference in least‐squares mean; MTPJ, metatarsophalangeal joint; OMERACT, Outcome Measures in Rheumatology.

#### Pressure time integrals

Table 5 presents the regression results comparing ultrasound synovitis with PTIs. Participants with moderate/severe synovitis at the first MTPJ displayed a significant reduction in PTIs beneath the hallux compared with those with none/minimal synovitis at this joint (*P* = 0.039). Participants with moderate/severe at the second MTPJ and fourth MTPJ also displayed significantly reduced PTIs beneath the lesser toes compared with those with none/minimal synovitis in these joints (*P* = 0.038 and *P* = 0.031, respectively). No other significant differences in peak plantar pressure were observed for synovitis in other joints.

**Table 5:** Difference in pressure time integrals (kPa*sec) between none/minimal synovitis and moderate/severe synovitis according to the combined EULAR/OMERACT scores*

	Grade	First MTPJ mean	Diff (95% CI)	*P*	Second MTPJ mean	Diff (95% CI)	*P*	Third MTPJ mean	Diff (95% CI)	*P*	Fourth MTPJ mean	Diff (95% CI)	*P*	Fifth MTPJ mean	Diff (95% CI)	*P*
Heel	None/minimal	64.10	9.92 (–8.15 to 28.00)	0.28	67.45	1.00 (–16.49 to 18.49)	0.91	73.96	–8.92 (–27.50 to 9.66)	0.34	68.44	–0.49 (–17.71 to 16.73)	0.96	59.87	11.28 (–7.87 to 30.42)	0.24
	Moderate/severe	74.03			68.45			65.04			67.95			71.15		
Midfoot	None/minimal	41.72	–4.26 (–14.23 to 5.71)	0.40	42.37	–4.23 (–13.36 to 4.90)	0.36	42.57	–3.60 (–13.38 to 6.19)	0.46	41.68	–2.68 (–11.57 to 6.21)	0.55	43.38	–4.63 (–15.06 to 5.81)	0.38
	Moderate/severe	37.46			38.14			38.97			39.00			38.75		
First metatarsal	None/minimal	63.83	–2.53 (–21.03 to 15.96)	0.79	65.76	–4.86 (–22.50 to 12.77)	0.58	61.51	1.82 (–16.86 to 20.50)	0.85	55.85	11.06 (–5.98 to 28.10)	0.20	62.82	0.59 (–19.03 to 20.20)	0.95
	Moderate/severe	61.29			60.90			63.33			66.91			63.41		
Second metatarsal	None/minimal	78.20	–11.16 (–24.84 to 2.53)	0.11	76.75	–5.34 (–18.60 to 7.93)	0.42	69.34	6.32 (–7.47 to 20.11)	0.36	72.69	0.49 (–12.48 to 13.46)	0.94	80.15	–8.65 (–23.21 to 5.90)	0.24
	Moderate/severe	67.04			71.42			75.66			73.18			71.50		
Third to fifth metatarsals	None/minimal	67.32	–3.22 (–13.53 to 7.09)	0.54	69.21	–5.62 (–15.59 to 4.36)	0.27	65.86	–1.11 (–11.52 to 9.30)	0.83	64.80	1.14 (–8.43 to 10.71)	0.81	68.83	–4.16 (–15.27 to 6.94)	0.46
	Moderate/severe	64.09			63.59			64.75			65.94			64.67		
Hallux	None/minimal	45.56	–13.20 (–25.71 to –0.69)	**0.039**	44.05	–6.48 (–18.93 to 5.98)	0.30	41.09	–1.80 (–14.51 to 10.91)	0.78	40.13	–0.46 (–12.61 to 11.70)	0.94	41.52	–3.02 (–16.69 to 10.65)	0.66
	Moderate/severe	32.36			37.57			39.29			39.68			38.50		
Lesser toes	None/minimal	25.45	–5.63 (–13.86 to 2.61)	0.18	28.14	–7.86 (–15.28 to –0.44)	**0.038**	21.31	2.44 (–5.30 to 10.19)	0.53	27.96	–7.60 (–14.48 to –0.72)	**0.031**	26.65	–3.01 (–11.55 to 5.53)	0.48
	Moderate/severe	19.83			20.28			23.75			20.36			22.64		

* *Source:* Results are presented adjusted for BMI. Bolded ***P*** values indicate significant differences in pressure time integrals between combined EUALR/OMERACT grades as *P* < 0.05. None/minimal synovitis = grade 0 or 1.

Moderate/severe synovitis = grade 2 or 3. Mean = least‐squares mean. Results are unadjusted for ethnicity.

Diff, difference in least‐squares mean; MTPJ, metatarsophalangeal joint; OMERACT, Outcome Measures in Rheumatology.

## DISCUSSION

Participants in the current study presented with clinical evidence of moderate foot disease activity, evident by palpable tenderness. However, the proportion of participants showing sonographic evidence of inflammation was much higher. This finding is consistent with previous research.^30^, ^31^ Furthermore, people with RA who are defined as being in remission still exhibit ultrasound evidence of persistent synovitis,^32^, ^33^ which is highly predictive of future flares and bone erosion in the small joints of the hands and feet.^34^, ^35^ This reinforces the usefulness of routine ultrasound to monitor inflammation and support timely interventions that reduce and prevent the inflammatory‐driven soft tissue and osseous changes seen in people with RA.

The results from this study demonstrated no direct association between plantar pressure and synovitis within the same region of the foot. However, participants who had sonographic evidence of synovitis in the second and fourth MTPJs walked with reduced PP and PTIs beneath the lesser toes, whereas those with first MTPJ synovitis walked with reduced hallux PTIs. These findings may be suggestive of an offloading strategy in which synovitis in the MTPJs results in reduced propulsion and use of the toes. This distribution of pressure is a common finding observed in people with RA and has been reported in combination with reduced ankle motion and overall flattening of the gait curve.^36^ Reduced lesser toe contact area, and therefore pressure during propulsion, has also been observed in people with RA, particularly in the presence of digital deformity.^4^ The presence of synovitis at the first MTPJ may be related to the frequency of structural damage, inflammation, and deformity (hallux valgus) observed at this joint in people with RA, which reduces first MTPJ function; therefore, the plantarflexory force exerted by the hallux during propulsion.^4^

The current results are consistent with the inverse correlation between pressure and sonographic pathology observed in previous studies.^10^, ^15^ Lower toe pressures have been observed with higher erosion scores in people with RA,^10^ and lower lateral forefoot pressures have been observed with ultrasound pathology (combined score of synovial hypertrophy and erosion),^15^ further supporting the hypothesis that people with RA may biomechanically adapt pressure away from areas of forefoot pathology. Further work is required to determine whether these offloading strategies may be more clinically important than raised plantar pressures in terms of impact on foot and lower limb pain and disability, particularly considering elevated plantar pressures are not significant predictors of foot ulceration in this population.^37^

The lack of association between site‐specific plantar pressure and synovitis may also be attributed to the lack of correlation between subclinical inflammation seen on ultrasound and clinically evident signs of inflammation, such as palpable tenderness and pain.^30^, ^31^ It may be that clinical pain and swelling influence ambulation and pressure distribution to a greater degree than subclinical pathology. However, research is yet to show a direct relationship between plantar pressure and foot pain within the same region of the foot^9^ or between plantar pressure and other measures of foot pain, swelling, and disability.^6^ These results suggest that other factors also influence the distribution of plantar pressure in people with RA.

The current study adds to the body of knowledge exploring the importance of plantar pressure assessment, which may be of value in directing the most appropriate treatment strategies in the presence of forefoot synovitis in people with RA. Current interventions for foot involvement in RA are largely focused on pressure reduction and include therapeutic footwear^38^ and customization of orthoses using various materials and modifications.^39^, ^40^ However, from a rehabilitation perspective, findings from the current study highlight the importance of further exploration into the capacity of the soft tissue and joint structures to accommodate changes in pressure because of offloading strategies. The addition of preventative exercise therapy in people with RA (including strengthening of the intrinsic and extrinsic foot muscles, joint range of motion exercises, and stretching) may benefit foot health in combination with footwear, orthoses, and pharmacological management. These foot and ankle specific exercises are recommended in the management of RA to improve mobility, improve balance, and reduce pain.^41^

This study should be considered in light of a number of strengths and potential limitations. Although the number of participants was small, the study was sufficiently powered to detect between group differences. The sample consisted predominantly of New Zealand Europeans, which is reflective of the epidemiology of RA in New Zealand but may limit generalizability of the results to other ethnic groups. Statistical efficiency was also achieved through the mixed‐models approach, which addressed the dependence between the different regions of the plantar foot and the repeated measures from right and left feet of each participant. This allowed information present in the covariance between the different plantar regions of the feet as well as between repeated right and left foot measures to be used. The analyses did not adjust for walking speed, which has been shown to impact plantar pressure.^42^ However, previous research has shown that walking speed does not impact the association between plantar pressure and disease activity.^6^ This study also considered the influence of sonographic pathology on plantar pressure at all locations of the plantar foot, not just the forefoot, as is common in other studies of people with RA.^6^ However, the presence of synovitis in other foot and lower limb joints was not considered, nor was the presence of inflammation in other soft tissue structures, which may also have an impact on pressure distribution during walking. It should also be noted that although intra‐reader reliability for both power Doppler and synovial hypertrophy were strong, the inter‐reader reliability for synovial hypertrophy was moderate. Although this is consistent with published reliability scores for ultrasound assessment of MTPJs,^43^, ^44^, ^45^, ^46^ it is possible that the between‐reader variability may have resulted in overestimation or underestimation of the final scores used in the analysis. A further limitation was the inability to determine the cause‐effect relationship between synovitis and plantar pressure because of the cross‐sectional design of the study. Finally, plantar pressure was captured only during barefoot walking, and it may be that pressure and friction from footwear, particularly in the presence of bony prominences and deformity,^47^ may contribute to synovitis and/or influence pressure distribution.

In conclusion, the results from this study have shown that people with RA frequently present with ultrasound evidence of MTPJ inflammation. Although no direct association was found between synovitis and pressure within the same regions of the foot, people with MTPJ synovitis exhibit reduced pressures beneath the toes. These findings may be suggestive of an inverse relationship between plantar pressure and soft tissue pathology, which is consistent with an offloading strategy and reduced use of the toes during propulsion.

## AUTHOR CONTRIBUTIONS

All authors were involved in drafting the article or revising it critically for important intellectual content, and all authors approved the final version to be published. Anderson, Ihaka and Dr. Stewart had full access to the data in the study and take responsibility for the integrity of the data and the accuracy of the data analysis.

### Study conception and design

Ihaka, Stewart.

### Acquisition of data

Anderson, Ihaka, Stewart.

### Analysis and interpretation of data

Anderson, Ihaka, Bowen, Dando, Stewart.

## Supporting information


Disclosure form:
